# Effects of ECM protein micropatterns on the migration and differentiation of adult neural stem cells

**DOI:** 10.1038/srep13043

**Published:** 2015-08-12

**Authors:** Sunghoon Joo, Joo Yeon Kim, Eunsoo Lee, Nari Hong, Woong Sun, Yoonkey Nam

**Affiliations:** 1 Department of Bio and Brain Engineering, Korea Advanced Institute of Science and Technology (KAIST), Daejeon 305-338, Republic of Korea; 2 Department of Anatomy and Division of Brain Korea 21, Korea University College of Medicine, Seoul 136-705, Republic of Korea

## Abstract

The migration and differentiation of adult neural stem cells (aNSCs) are believed to be strongly influenced by the spatial distribution of extracellular matrix (ECM) proteins in the stem cell niche. *In vitro* culture platform, which involves the specific spatial distribution of ECM protein, could offer novel tools for better understanding of aNSC behavior in the spatial pattern of ECM proteins. In this work, we applied soft-lithographic technique to design simple and reproducible laminin (LN)-polylysine cell culture substrates and investigated how aNSCs respond to the various spatial distribution of laminin, one of ECM proteins enriched in the aNSC niche. We found that aNSC preferred to migrate and attach to LN stripes, and aNSC-derived neurons and astrocytes showed significant difference in motility towards LN stripes. By changing the spacing of LN stripes, we were able to control the alignment of neurons and astrocytes. To the best of our knowledge, this is the first time to investigate the differential cellular responses of aNSCs on ECM protein (LN) and cell adhesive synthetic polymer (PDL) using surface micropatterns. Our findings would provide a deeper understanding in astrocyte-neuron interactions as well as ECM-stem cell interactions.

Adult neural stem cells (aNSC) are self-renewing, multipotent cells, that can be found in such specialized niches as the subventricular zone (SVZ) of lateral ventricle and subgranular zone (SGZ) of hippocampus in the adult mammalian brain^1^. The fate of aNSC is regulated by various signalling cues in the stem cell niche. For example, NSCs prefer to adhere to the adjacent basal lamina of blood vessels, where the extracellular matrix (ECM) such as laminin (LN), fibronectin and collagen are abundant^2^. It is also known that the spatial distribution of ECM controls migration and differentiation through the ECM/receptor signalling^3^. However, due to the limitation of *in vivo* experiments, the key parameter for ECM patterning to regulate the migration and differentiation of aNSC is poorly understood. Thus, the techniques to elucidate the mechanism of interaction between various cues and aNSCs are needed.

Engineered culture substrates have been introduced to investigate cellular responses *in vitro* by systematically controlling cellular environments. Recently, there were many researchers who modified physical and chemical properties of the entire culture substrate to study the response of neural stem cells on the engineered culture substrate. Physical cues such as stiffness^4^,^5^, the nanotopography made by nanofibers^6^, and the microgroove by PDMS substrate^7^ affected neuronal differentiation of neural stem cells. On the other hand, the spatial control of biofunctional proteins at the microscale on the culture substrate have become available owing to the development of microfabrication techniques such as microcontact printing (μCP)^8^,^9^,^10^,^11^,^12^, microstenciling^13^,^14^, and microfluidic channel^15^,^16^. The biofunctional protein patterned culture substrates were also used as a tool for studying the interaction between cellular response of neural stem cells (NSCs) and specialized microenvironment at the cellular scale. The NSC patterning method based on pre-patterned polypeptides or ECM protein by μCP was reported for observing the cellular response like morphology, migration and neuronal differentiation on the confined area^17^,^18^. Konagaya *et al*. reported NCS responses on the substrate patterned with five different growth factors by photo-assisted patterning^19^. To study the migration and differentiation of NSC, Wang *et al*. developed a culture substrate which combined two distinct signaling proteins^20^. So far, there has been no study that observed the response of aNSCs on the various distributions of ECM proteins using engineered culture substrates even though the spatial distribution of ECM proteins in the stem cell niche plays important roles in the neural differentiation of aNSCs.

In this work, we introduce an ECM protein patterning chip for studying the migration and differentiation of aNSC on the various spatial distribution of ECM proteins. For the first time, we applied an engineered culture substrate to evaluate differential responses of neurons and astrocytes from aNSC using the competition assay. We chose the LN as the ECM protein that was highly found in the basal lamina. By using μCP, LN stripes, which had four different spacings, were patterned on PDL-coated cell adhesive surface. The migration and differentiation of aNSCs on the substrate were quantitatively analyzed by immunocytochemistry and live imaging. We found that the motility on the PDL background was cell-type dependent, and the different motilities of aNSC-derived neurons and astrocyte made unique spatial separation of two progeny cell types. The μCP of functional proteins on the PDL-coated substrates is an easily implementable method for producing culture substrates that is intended to spatially segregate different cell types based on their substrate affinity.

## Results

### Fabrication and characterization of LN stripes on PDL coated substrate

**Figure 1:**
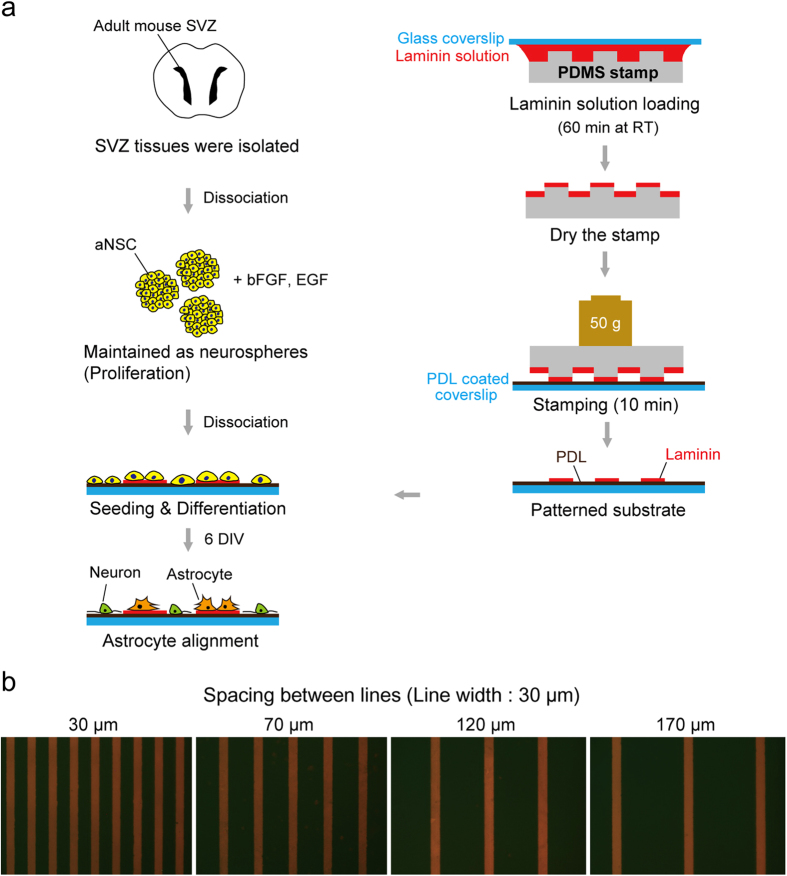
Fabrication of a micro-patterned LN/PDL substrate. (**a**) Schematic illustration of the experiment to involve micro-contact printing of laminin (LN) on poly-D-lysine (PDL) coated glass coverslip and culturing aNSCs. (**b**) Fluorescence micrographs of 4 types of LN stripes on the PDL coated glass substrate. Four types of LN stripes were designed to contain the stripes with 30 μm of width and 30, 70, 120, 170 μm of spacing, respectively.

Four types of LN stripe patterns were designed with an identical line width (30 μm), but with different spacings: 30, 70, 120 and 170 μm. The line width was chosen to be comparable to a single cell size, and spacing was varied to test the migration and differentiation of aNSCs on under different spatial distributions of ECM proteins. In order to ensure the attachment of the LN stripes on to the PDL coated substrate, we made an ‘ink’ composed of LN solution and fluorescence antibody (1:1000). Fig. 1b shows fluorescence micrographs of 4 types of LN stripes on PDL coated glass substrates. According to the fluorescence images, LN stripes were successfully immobilized on PDL surfaces. Due to the initial PDL coating, the entire surface, including on-pattern and off-pattern, was cell-adhesive.

### Astrocyte alignment to stamped LN lines

**Figure 2:**
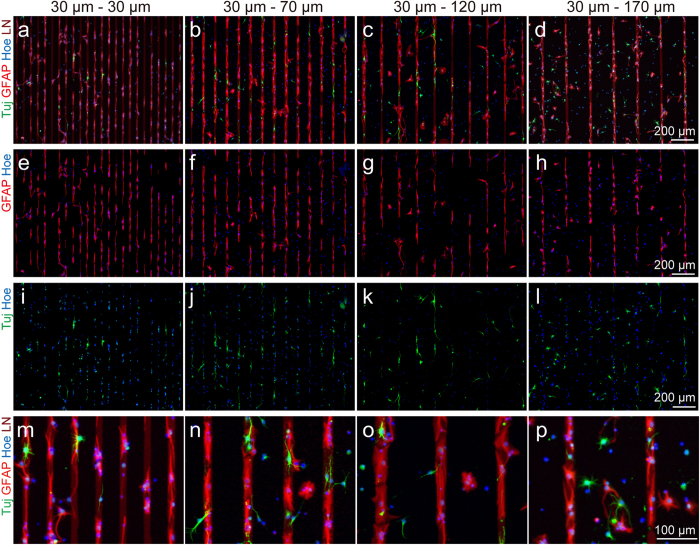
Differentiation and alignment of aNSCs on LN patterned culture substrate. (**a-d**) Fluorescence micrographs of GFAP, Tuj-1, and nucleus stained aNSC at 6 DIV on the LN stripe patterns with 30, 70, 120, 170 μm of spacing. (**e-h**) Fluorescence micrographs of GFAP and nucleus stained aNSC at 6 DIV on the LN stripe patterns with 30, 70, 120, 170 μm of spacing. (**i-l**) Fluorescence micrographs of Tuj-1 and nucleus stained aNSC at 6 DIV on the LN stripe patterns with 30, 70, 120, 170 μm of spacing. (**m-p**) Fluorescence micrographs at high-magnification of GFAP, Tuj-1, and nucleus stained aNSC at 6 DIV on the LN stripe patterns with 30, 70, 120, 170 μm of spacing.

In order to find out the patterns of migration and differentiation of aNSC on the designed substrate, we seeded aNSCs on the patterns and promoted spontaneous differentiation by removing growth factors. After 6 day *in vitro* (DIV), the cells were fixed, and subsequently immunostained with Tuj-1 (marker for neuron), and glial-fibrillary acidic protein (GFAP; marker for astrocyte). Figure 2 shows representative fluorescence micrographs of differentiated aNSCs that grow on the 4 differently patterned substrates; under all conditions, aNSCs were readily differentiated into both neurons and astrocytes. In all spacing conditions, astrocytes were mainly found on LN stripes and there was much less astrocytes present in PDL background. On the other hand, neurons were highly localized in narrow spacings, but the alignment to LN stripe was weakened as the spacing increased.

**Figure 3:**
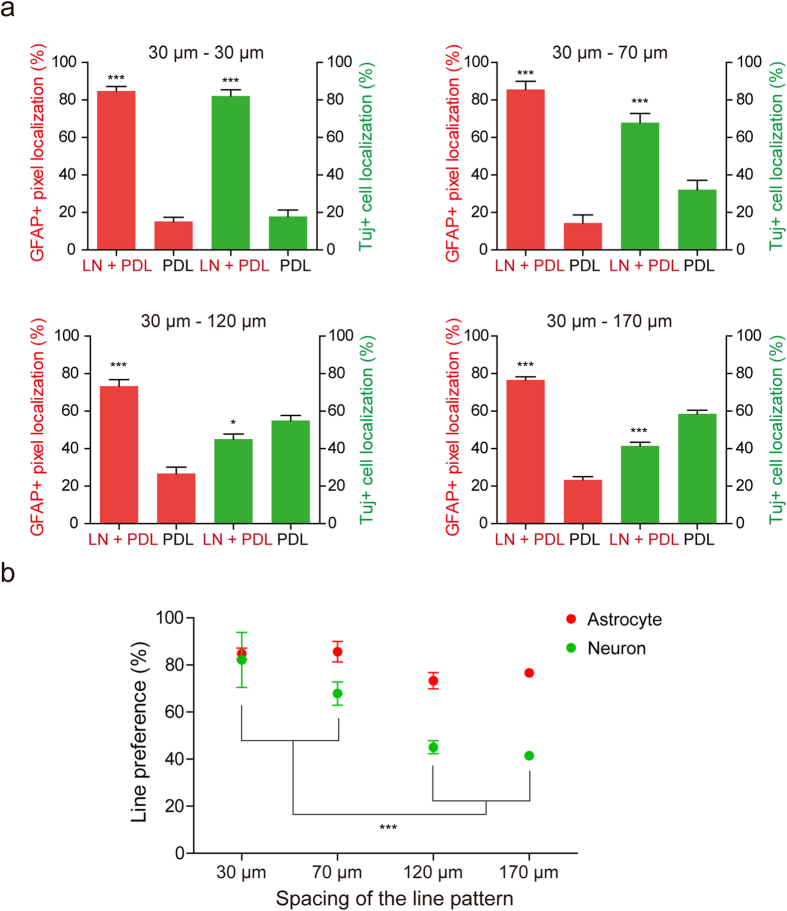
The qualification of the location of neurons and astrocytes derived from aNSCs at 6 DIV on the pattern. (**a**) GFAP positive pixels and Tuj-1 positive cells alignment for each substrate (mean ± S.E., ***p < 0.001). (**b**) The line preference of neurons and astrocytes derived from aNSCs at 6 DIV for each patterns substrate (mean ± S.E. n = 12, 9, 13 and 19 for 30, 70, 120 and 170 μm, respectively, ***p < 0.001).

To quantify the degree of alignment on LN lines, we calculated the percentage of Tuj-1 positive cells or GFAP positive cells located on the LN line (LN + PDL) and background (PDL), respectively (Fig. 3a). The percentage of GFAP positive pixels localized on LN line on all kinds of spacing, indicated in Fig. 3, was significantly higher compared to pixels on localized on background (84.8 ± 6.4% on 30 μm–30 μm; 85.6 ± 4.3% on 30 μm–70 μm; 73.3 ± 3.4% on 30 μm–120 μm; 76.6 ± 1.7% on 30 μm–170 μm, mean ± S.E., p < 0.001). In the case of neuronal distribution, for 30 μm–30 μm and 30 μm–70 μm patterns, the percentage of Tuj-1 positive cells localized on the LN line was significantly higher than that on the background (82.1 ± 3.4% and 67.8 ± 5.0% respectively, p < 0.001). On the other hand, the percentage of Tuj-1 positive cells localized on LN line was lower than that on the background of 30 μm–120 μm and 30 μm–170 μm patterns (45.0 ± 2.8% and 41.5 ± 1.9% respectively, mean ± S.E.). Comparisons of line preferences showed statistically significant preferences of astrocytes to LN lines in all spacings vs. spacing length-dependent reduction of preferences of neurons to LN lines (Fig. 3b).

### Migration of aNSC on the engineered culture substrate in early stage

**Figure 4:**
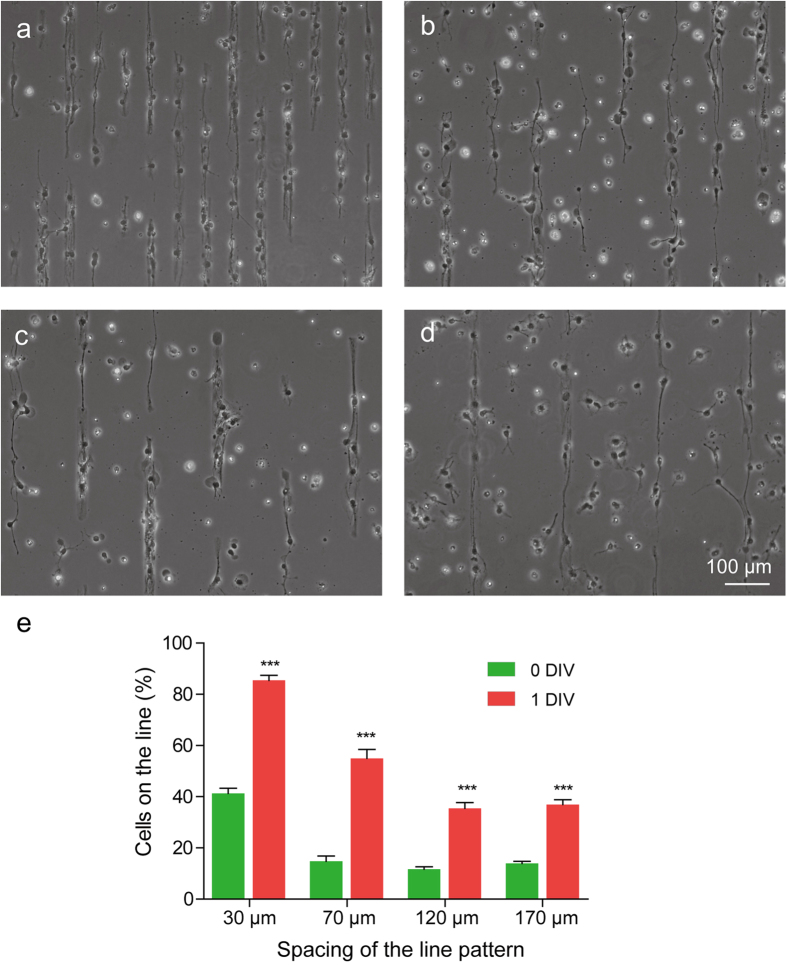
The migration of aNSC on the engineered culture substrate in early stage. (**a-d**) Phase-contrast micrographs of aNSC on the LN stripe patterns with 30, 70, 120, 170 μm of spacing at 1 DIV. Even though LN stripes were not indicated, the stripes were identifiable owing to the preferential localization of aNSCs on LN stripes. (**e**) Distribution of cells on LN line pattern at 0, 1 DIV (mean ± S.E., n = 10, *p < 0.001).

In order to address the mechanism of the cell type-dependent responses to the spacing of stripes, we compared the cells in LN stripes at two different time points (1 hour vs. 24 hours after seeding). Figure 4 shows phase contrast images of aNSCs on 4 groups (spacing: 30 μm, 70 μm, 120 μm and 170 μm) at 1 DIV. One hour after initial cell seeding (0 DIV), the percentage of cells on LN line was 41.3 ± 1.9% (30 μm spacing), 14.8 ± 2.0% (70 μm spacing), 11.7 ± 1.0% (120 μm spacing), and 14.0 ± 0.8% (170 μm spacing), and the percentages sharply increased to 85.5 ± 1.9% (30 μm spacing), 55.0 ± 3.5% (70 μm spacing), 35.5 ± 2.2% (120 μm spacing) and 37.0 ± 1.8% (170 μm spacing) after 24 hours (1 DIV) (Fig. 4e). For all patterns, the percentage of cells on LN line at 1 DIV was significantly higher than that at 0 DIV. This result indicates that aNSCs had a preference for LN lines compared to the PDL substrate. However, there was a significant trend that more cells remained on the gap between the LN lines by increasing the length of spacing, presumably due to the limited migratory capacity of cells towards LN stripes during the given time overall. These results suggest that line preferences of neurons or astrocytes in the previous section could be affected by the early-phase behavior of aNSCs on the LN patterns.

### Differential migration between neurons and astrocytes derived from aNSCs

**Figure 5:**
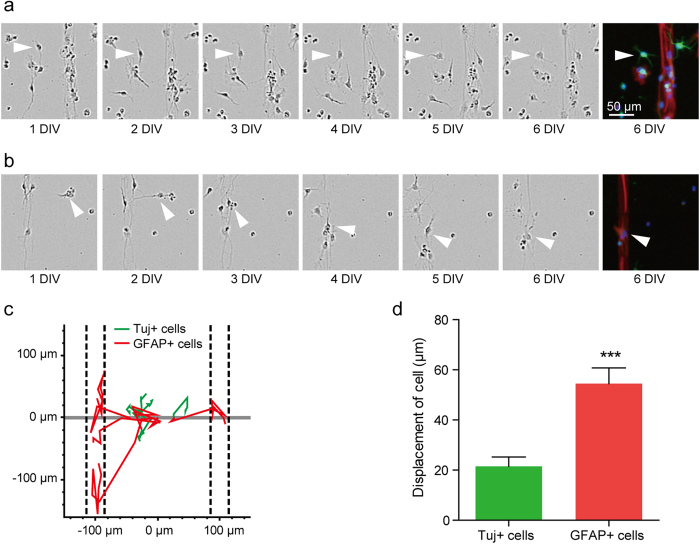
Tracking aNSCs, which were placed on PDL background initially for 6 days. (**a**) Phase-contrast micrographs of Tuj-1 positive cell at 1 to 6 DIV and fluorescence micrographs of Tuj-1 positive cell at 6 DIV. (**b**) Phase-contrast micrographs of GFAP positive cell at 1 to 6 DIV and fluorescence micrographs of GFAP positive cell at 6 DIV. The white arrowhead indicates the target cell. (**c**) Traces of 5 Tuj-1 positive cells (green) and 5 GFAP positive cells (red) on the substrate. The dashed lines mean the location of LN stripes. (**d**) Average displacement (mean ± S.E., n = 22 and 20 for Tuj-1 positive cells and GFAP positive cells, respectively) of the Tuj-1 positive cells and GFAP positive cells for 6 days. (***p < 0.001).

Although aNSCs’ preference for the LN line may explain the strong alignment of all progeny cells on the 30 μm spacing pattern, the effect of the width of line spacing on the selective displacement of neurons for LN line is unclear yet. To investigate the impact of cell behavior during differentiation on the patterned substrates, we tracked the cells for 6 days after seeding. At the end of the experiment, the samples were stained with Tuj-1 and GFAP to identify the type of cells. Figure 5a,b show the phase contrast images of aNSC on the LN stripes with PLL background (30 μm width and 170 μm spacing) for 6 days and fluorescent images of the same field. Figure 5a shows time-lapse images of an aNSC-derived neuron on the background (PDL). While the neuron on the PDL background made very little movement for 6 days, an aNCS-derived astrocyte migrated onto LN line in 3 days, and it stayed on the LN stripe (Fig. 5b). Figure 5c shows five migration traces for each cell type. All astrocytes successfully migrated to the LN lines (indicated by the dashed lines) and dwelled on them, while neurons did not show any noticeable migratory behavior towards the LN line. Figure 5d shows the average displacement of neurons and astrocytes, which were on the PDL area initially. The average displacement of astrocytes was 54.5 ± 6.3 μm (mean ± S.E., n = 22). It was significantly larger than that of neurons (21.5 ± 3.7 μm, n = 20). Therefore, neurons differentiated from NSCs on the PDL background would often fail to migrate onto the LN stripe, and more neurons would stay on PDL background with the increase in spacing.

### Differentiation of aNSCs on laminin coated substrate

**Figure 6:**
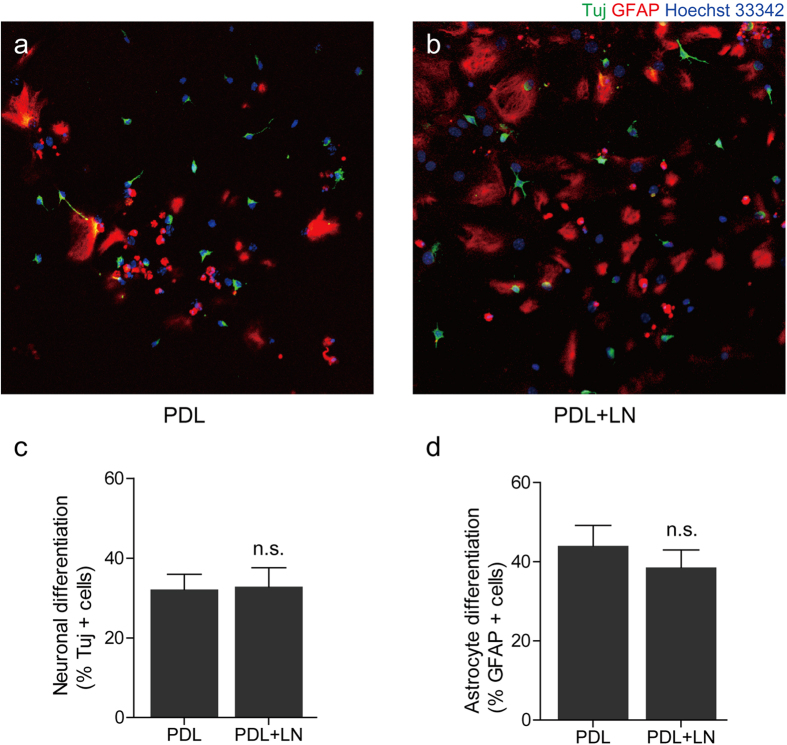
The differentiation of aNSC on laminin coated substrate. (**a**) Fluorescence micrograph of aNSCs on PDL coated substrate at 6 DIV. (**b**) Fluorescence micrograph of aNSCs on PDL and LN coated substrate at 6 DIV. (**c**) Neuronal differentiation rate (mean ± S.E. n = 10) of aNSCs on each substrate. (**d**) Astrocyte differentiation rate (mean ± S.E. n = 10) of aNSCs on each substrate. n.s.: not significant.

Next, we performed a control experiment to check if LN/PDL or PDL substrates had an influence on the differentiation of neurons or astrocytes from aNSCs. aNSCs were cultivated on two different substrates independently: PDL substrates with and without LN coating. The substrate coated with both PDL and LN (PDL+LN group) was a model substrate for LN lines stamped on PDL, and the substrate coated with PDL only (PDL group) was a model substrate for the PDL background. Figure 6a,b show fluorescence images of aNSCs on each group at 6 DIV. The percentage of neurons (Tuj+ cells) and astrocytes (GFAP+ cells) were measured. There was no statistical difference in neuronal or astrocytic differentiation from aNSC cultured on two different surface conditions (Fig. 6c). Accordingly, we concluded that it was not likely that aNSC differentiation was the critical factor in the observed line preferences of LN stripes or PDL background.

## Discussion

**Figure 7:**
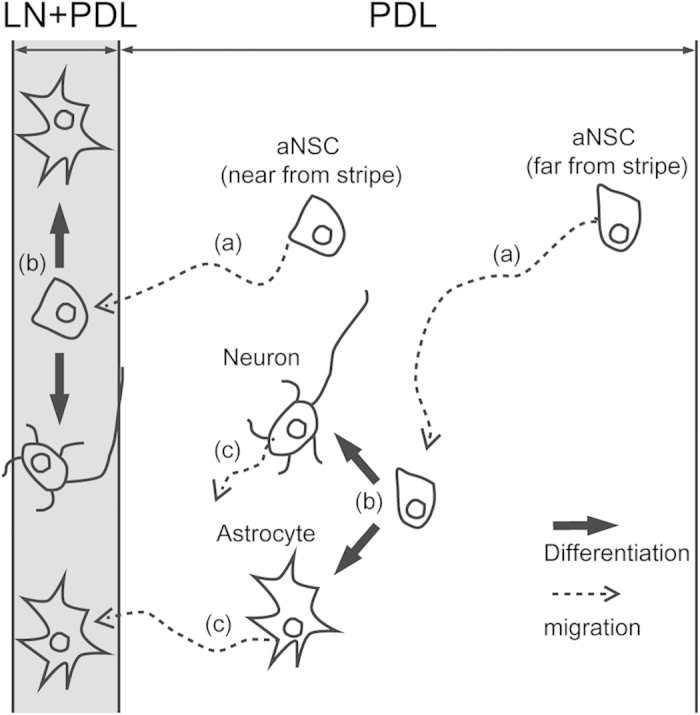
Schematic diagram of astrocyte aligning procedures. (**a**) aNSCs on PDL background were motile. aNSCs where was near from LN stipe migrated to the stripe. However, aNSCs was far from LN stripe could not reach the stripe. (**b**) aNSCs on both LN stripe and PDL background differentiated to neurons and astrocytes with similar probability. (**c**) The astrocytes were more motile than the neurons on PDL background. Some astrocytes near from stripe migrated to LN stripe.

In this work, micropatterned LN stripe patterns have been applied to study the migration and differentiation of aNSCs. The culture substrate, which had LN stripes on the PDL background, was used for competition assay between LN and PDL. It was evident that there were more astrocytes present on LN stripes compared to PDL background. The LN preference of astrocytes at 6 DIV was independent of the line spacing (30 ~ 170 μm). In contrast, neuronal cells preferred for the LN reduced with the increase in line spacing. When the spacing was 120 μm or 170 μm, there were more neurons located at PDL area (Fig. 3). We reasoned that there was a difference in cell migration on LN and on PDL surfaces. Initially, when we cultured aNSCs on our substrates, aNSCs were randomly distributed on the entire substrate regardless of the LN line patterns. We found that aNSCs more actively migrated on the PDL surfaces. When aNSCs were near the LN areas, almost every cell migrated onto the LN (Fig. 4). The aNSCs located on the LN stripes did not move outside the line. Considering the expression of laminin receptors by aNSC, it is likely that the interaction between the receptor and laminin forced the displacement of aNSCs onto the LN stripes^21^. Even though the PDL layer was also cell adhesive, LN stripes on the PDL coated substrate were more effective in attracting aNSCs. After differentiation, neurons and astrocytes showed different cell migratory behaviour on the PDL surfaces. Neurons were much less migratory than astrocytes on the PDL surfaces. The mean migration length was 2 ~ 3 times longer for astrocytes (21.5 μm vs. 54.5 μm) (Fig. 5). As a result, aNSC-derived astrocytes were much more localized on LN stripes. Figure 7 summarizes current observations and possible mechanisms underlying this phenomenon.

Controlling the differentiation of neural stem cells into specific cell types such as neuron, oligodendrocytes, or astrocytes are of interest in neural tissue engineering. Neural stem cell differentiation is known to be regulated by molecular signaling pathways such as Platelet-derived growth factor (PDGF), fibroblast growth factor (FGF), bone morphogenetic protein (BMPs), Notch ligand and cytokines^22^,^23^,^24^,^25^. Neuronal or glial differentiation in the developing CNS is regulated by several basic helix-loop-helix (bHLH) transcription factors, such as oligo1 and oligo2 for glial specification^26^,^27^, and neurogenin1 and 2 (Ngn1 and Ngn2), Mash1, and Math1 for neuronal differentiation^28^,^29^,^30^. It has been shown that physical cues, such as stiffness or surface topography, could be used to regulate neural stem cell differentiation. These works mainly investigated the efficiency of such cues in differentiating NSCs into neurons or astrocytes. On the various stiffnesses of culture substrates, neural differentiation and astrocyte differentiation were activated on softer substrate. On the other way, the differentiation of oligodendrocyte was favored on harder substrate^4^,^5^. Neuronal differentiation was promoted on nano-topography which was fabricated by alignment of polystyrene fibers with 10–1000 nm in diameter^6^. Our work is one of a few works that studied cell differentiation on surface-bound micropatterns using ECM proteins. The astrocyte preference to 30-μm LN/PDL lines was mainly due to cell migration behavior. In Fig. 6, we were not able to find any significant difference of neuron and astrocyte ratio on LN/PDL or PDL substrates. In addition, there were no significant differences in line preferences between astrocytes and neurons for 30–30 and 30–70 chips. If 30-μm LN/PDL line had higher efficiency in differentiating aNSCs into astrocytes, 30–30 and 30–70 in Fig. 3 could not be obtained. Thus, our chip design suggests that it we can obtain spatial differentiation effects through different cellular responses to LN/PDL and PDL. But, it is intriguing to find a way to control neuron and astrocyte differentiation by solely restricting cell geometry through surface-bound micropatterns. If we can control neural stem cell fate by simply restricting cell shape, it would be powerful platform for neural tissue engineering.

We tried to create a similar microenvironment using μCP, and it is well known that the micro-niche in the adult neurogenic regions provides on environment for the maintenance and differentiation of aNSCs^2^,^31^. One of the important substrates affecting NSC functions is the basement membrane of blood vessels which are composed of many ECM molecules including LN^32^,^33^. Interestingly, one end-feet of aNSC is in close contact with LN-rich blood vessels, and the perturbation of this aNSC-vessel interaction alters the proliferation of aNSCs^21^. Blood vessels produce linear and network-like architecture, but the significance of network-like placement of LN substrates for the aNSC function is not as well understood. To mimic this specialized structure, we generated linearly patterned biomolecules at microscale, and observed the response of aNSC on the substrate patterns. To produce selective biomolecule stripe patterns on the substrate, microfluidic channels have been used as the conventional technique^34^. However, compared to conventional method, our approach using μCP can produce various patterns (e.g. circles, dots), which give engineered culture substrates with higher geometrical variations. Furthermore, since μCP allows migration of cells between two different surfaces, it provides unique opportunities to monitor cellular choice of their placement. Although LN was the only substrate used in the current study, there are many bioactive molecules that can be used for further studies according to many reports about patterning neural signalling proteins such as fibronectin, netrin-1, ephrinA5, L1 and N-cadherin by μCP^20^,^35^,^36^,^37^. Our approach of making selective bioactive molecule patterns on the PDL coated substrate using μCP may be powerful for studying the response of not only an aNSC but also many other cell types.

During the development, neurons and astrocytes derived from the same NSC populations behave differently. For example, neurons produced from SVZ NSCs exclusively migrate towards the olfactory bulb via rostral migratory stream. On the other hand, astrocytes produced from the same NSC populations migrate radially and contribute to the astrocytes in the forebrain^38^,^39^,^40^. In addition, it is known that neurons and glial cells from NSCs are differentially positioned in response to the brain injury^41^. Especially, glial cells often form scar near the injury penumbra which perturb long-term regeneration of axons^42^. In this respect, differential migratory responses of neurons in contrast to astrocytes to the ECM substrates, including LN, could provide a fundamental framework for understanding how brain cytoarchitectures are remodeled during the brain regeneration, and our patterned culture system may provide a novel method to explore the biological significance of the ECM substrates *in vitro*. Moreover, this is the first time observing the cellular responses of aNSCs on ECM protein (LN) and cell adhesive synthetic polymer (PDL), respectively by competition assay. Responses of aNSCs and the derived cells to the varying spacing of patterns are worthwhile to consider when designing biomaterials in tissue engineering field that using aNSCs.

In this work, we proposed a new approach to study migration and differentiation of aNSCs by use of engineering technology. To the best of our knowledge, this is the first investigation that compares the differential cellular responses of aNSCs on ECM protein (LN) and cell adhesive synthetic polymer (PDL) by competition assay. On this patterned substrate, it was discovered that undifferentiated aNSCs and the astrocytes that were derived from aNSCs migrated towards the LN stripes in favor of the PDL background. However, the neurons derived from aNSCs on the PDL background did not move to the LN stripes, allowing the control of spatial separation rate of astrocyte and neurons. These findings could be used as a groundwork in designing astrocyte-aligned-cell-chips that may further serve as the platform for the *in vitro* analysis of astrocyte–neuron interaction and related neural tissue engineering.

## Methods

### Substrate Preparation

We fabricated polydimethoxysiloxane (PDMS) stamps by soft-lithography. A SU-8, negative photoresist, was patterned by photolithography on a silicon wafer as the master for molding PDMS microstamps. We used the SU-8 2010 (Microchem, USA) to obtain a mold with a size of 20 μm in height. The SU-8 master was coated with trichloro(1*H*,1*H*,2*H*,2*H*-perfluorooctyl)silane (Sigma-Aldrich, USA) using vapor deposition method to facilitate the release of PDMS from the SU-8 master. The mixture of PDMS prepolymer and the curing agent (10 : 1 (w/w), Sylgard 184 silicon elastomer kit, Dow Corning Corp., USA) was poured on the SU-8 mold and cured for 5 hours at 60 °C in the convection oven. The cured PDMS was gently removed and cut into small pieces (1 × 1 cm) for microstamps. Dust particles on the patterned side of PDMS stamps were removed with an adhesive tape. A glass coverslip (18 mm in diameter, Marienfield, Germany) was cleaned with acetone, IPA, and DI water for 5 min sequentially in an ultrasonic bath. Before PDL coating on the glass substrate, the coverslip was treated with an air-plasma (30 W, 0.7 Torr, Femto Science, Korea) for 1 min. Immediately, a drop of PDL solution (0.1 mg ml^−1^ in DI, MW 30,000–70,000, Sigma-Aldrich, USA) was applied on the coverslip for 2 hours at room temperature. Then, the coverslip was rinsed in running DI water and compressed dry air. For sterilization, the coverslip was sterilized with 70% ethanol (1 ml) for 1 min. Sterilized coverslips were dried, and left in the hood until LN stamping. Fig. 1a shows the fabrication process of laminin (LN) line pattern on a poly-D-lysine (PDL) coated glass coverslip. LN solution (30 μg ml^−1^ in PBS) was loaded on the pattern side of PDMS stamp for 1 h. For spreading the solution on the stamp, a bare glass coverslip was placed on the LN solution. After 1 h, the LN solution was removed, and the PDMS stamp was sequentially washed by PBS and DI water. Compressed air dried PDMS stamp was placed on a PDL coated glass coverslip with the patterned side down, and a weight of 50 g was applied for 10 min in the laminar flow hood. We used micropatterns with the following specifications: 30 μm in width, and 30 μm, 70 μm, 120 μm, and 170 μm, respectively in spacing.

### Adult neural stem cell culture

Adult male C57BL/6 (8–9 weeks-old) mice were obtained from ORIENT BIO (Seongnam, Korea). Adult mouse were sacrificed and the brain was isolated. After making 1-mm brain slices, the Subventricular zone (SVZ) tissues were isolated and incubated with digestion buffer including 0.8% papain (Worthington, Lakewood, NJ, USA) and 0.08% dispase II (Roche Applied Science) in HBSS for 45 min at 37 °C)^43^. Dissociated cells were seeded and maintained as neurospheres in DMEM/F12 media containing 1% N2, 2% B27, and penicillin-streptomycin. To keep aNSCs, growth factors containing basic fibroblast growth factors (bFGF, 20 ng/ml, Invitrogen) and epidermal growth factors (EGF, 20 ng/ml, Invitrogen) were added. To achieve the differentiation of the dissociated aNSCs, neurospheres were gathered and incubated with Accutase (Innovative Cell Technologies) for 5–10 min at 37 °C. Next, dissociated cells were seeded to LN patterned coverslips (diameter: 18 mm) and maintained without growth factors for 6 days. All experiments were performed in accordance with the guidance of the Institutional Animal Care and Use Committee (IACUC) of Korea Advanced Institute of Science and Technology (KAIST) and all experimental protocols were approved by IACUC of KAIST.

### Immunocytochemistry

For immunocytochemistry, cultured adult neural stem cells were fixed with 4% paraformaldehyde for 10 min at RT (room temperature). The samples were washed twice with PBS (phosphate buffer saline, pH 7.4, Gibco). To permeabilize cells and block nonspecific binding, the samples were treated with 0.1% Triton X-100 (Sigma-Aldrich) and 3% bovine serum albumin (BSA, Sigma) in PBS for 2 hours at RT. Then, the samples were reacted at 4 °C overnight with diluted primary antibodies in PBS with 0.1% Triton X-100 and 3% BSA. The following dilutions of primary antibodies were used: for neuron staining, anti-Tuj-1 (1:500; Sigma), and for astrocyte staining, anti-glial fibrillary acidic protein (GFAP, 1:1000; Sigma). After rinsing 3 times in PBS, the samples were treated for 1 hour at RT with appropriate secondary antibodies: Alexa Fluor^®^ 594-conjugated (1:1000, Invitrogen) or Alexa Fluor^®^ 488-conjugated (1:500, Invitrogen). Nuclei were labeled with Hoechst 33342 (1:1000, Sigma). The stained samples were mounted on a slide glass with Faramount (Dako, Denmark).

### Instruments and Characterizations

Fluorescent and phase contrast micrographs were taken from an inverted microscope (IX71, Olympus, Japan) that was equipped with a CCD microscope camera (DP71, Olympus). Live-cell imaging was performed with an IncuCyte ZOOM microscope (Essen Bioscience, USA). MATLAB (Mathworks) was used to analyze the distribution of GFAP positive pixels. From the micrographs, the number of cells on LN line and outside was counted with ImageJ software (NIH).

### Data analysis

To calculate the location of neurons, we counted the number of cells on LN stripes and PDL background in a single micrograph. To quantify the distribution of GFAP, we counted the number of pixels that was GFAP positive in a single micrograph. Data were represented by mean ± standard error (S.E.). Statistical significance was evaluated by one-way analysis of variance (ANOVA) followed by Bonferroni’s test for multiple comparisons and an unpaired Student’s two-tailed *t*-test (significant for *p* < 0.001) for two groups using Prism 5 (Graphpad Software, CA, USA).

## Additional Information

**How to cite this article**: Joo, S. *et al*. Effects of ECM protein micropatterns on the migration and differentiation of adult neural stem cells. *Sci. Rep*. **5**, 13043; doi: 10.1038/srep13043 (2015).
